# Supplementary material for: Efficacy and Safety of the Cudrania tricuspidata Extract on Functional Dyspepsia: A Randomized Double-Blind Placebo-Controlled Multicenter Study
Source: J Clin Med. 2021 Nov 16;10(22):5323. doi: 10.3390/jcm10225323 (PMC8621513; doi:10.3390/jcm10225323)
Supplement: Supplementary file 1 [file jcm-10-05323-s001.zip › jcm-1413954-supplementary.pdf]

Table S1. The frequency scales of the Nepean Dyspepsia Index

		Intervention group *n* = 39	P-value*	Control group *n* = 44	P-value*	p-value**	p-value†
Pain in the upper abdomen	Baseline	5.77±3.25		4.93±3.32		0.324	
	Day 28	3.23±2.50	<0.001	4.09±3.58	0.042	0.001	0.006
	Day 56	1.82±2.13	<0.001	4.05±3.70	0.059	<0.001	<0.001
Discomfort in the upper abdomen	Baseline	5.62±3.26		5.34±2.98		0.722	
	Day 28	2.92±2.52	<0.001	5.02±3.25	0.425	<0.001	<0.001
	Day 56	1.74±2.28	<0.001	4.55±3.54	0.065	<0.001	<0.001
Burning	Baseline	5.85±4.06		5.18±3.13		0.490	
	Day 28	3.03±3.00	<0.001	4.70±3.68	0.286	<0.001	<0.001
	Day 56	1.44±1.98	<0.001	4.55±3.27	0.207	<0.001	<0.001
Heartburn	Baseline	4.13±3.47		3.34±3.19		0.254	
	Day 28	2.00±2.35	<0.001	2.95±3.58	0.458	0.022	0.029
	Day 56	0.64±1.33	<0.001	3.18±3.35	0.752	<0.001	<0.001
Cramps	Baseline	3.08±3.34		2.70±3.17		0.621	
	Day 28	1.18±2.22	<0.001	2.80±3.45	0.820	0.002	<0.001
	Day 56	0.64±1.35	<0.001	2.59±4.02	0.822	0.002	0.004
Chest discomfort	Baseline	3.59±3.49		3.36±2.82		0.955	
	Day 28	1.97±2.28	0.002	3.16±3.20	0.562	0.032	0.007
	Day 56	0.85±1.42	<0.001	3.27±3.85	0.853	0.002	0.002
Inability to finish a regular meal	Baseline	4.56±3.44		4.41±3.91		0.853	
	Day 28	2.05±2.54	<0.001	3.95±3.60	0.402	0.002	0.028
	Day 56	0.85±1.69	<0.001	4.14±3.97	0.683	<0.001	<0.001
Bitter-tasting fluid in the mouth	Baseline	5.28±4.01		4.93±3.31		0.836	
	Day 28	2.59±2.81	<0.001	4.43±3.53	0.180	<0.001	0.003
	Day 56	1.05±1.99	<0.001	4.36±3.89	0.207	<0.001	<0.001
Fullness after	Baseline	7.56±3.02		7.75±2.79		0.765	

eating	Day 28	4.49±2.46	<0.001	6.73±2.97	0.010	0.002	0.002
	Day 56	3.18±2.08	<0.001	6.84±3.35	0.053	<0.001	<0.001
Pressure in the upper abdomen	Baseline	4.36±3.67		3.80±3.64		0.459	
	Day 28	2.05±2.33	<0.001	3.80±3.75	1.000	0.001	0.018
	Day 56	1.15±1.65	<0.001	4.30±3.76	0.224	<0.001	<0.001
Bloating	Baseline	6.05±3.46		6.52±3.86		0.425	
	Day 28	3.87±2.49	<0.001	6.52±3.87	1.000	0.004	0.005
	Day 56	2.28±2.36	<0.001	6.27±4.09	0.620	<0.001	<0.001
Nausea	Baseline	3.69±3.19		3.30±3.69		0.444	
	Day 28	1.56±2.64	<0.001	2.77±3.54	0.174	0.015	0.100
	Day 56	0.46±1.14	<0.001	2.70±3.53	0.194	<0.001	<0.001
Belching	Baseline	5.90±3.40		5.05±3.66		0.283	
	Day 28	3.18±2.90	<0.001	4.70±3.64	0.435	<0.001	0.001
	Day 56	2.31±2.45	<0.001	4.41±3.96	0.167	<0.001	<0.001
Vomiting	Baseline	1.59±2.88		1.07±2.12		0.667	
	Day 28	0.46±1.29	0.011	0.77±1.67	0.368	0.261	0.088
	Day 56	0.21±0.73	0.004	1.25±2.54	0.684	0.063	0.006
Bed breath	Baseline	2.08±2.99		2.80±3.30		0.302	
	Day 28	0.77±1.46	0.002	2.23±3.13	0.070	0.275	0.009
	Day 56	0.36±0.96	<0.001	2.41±3.73	0.403	0.102	0.013

*: Compared within group; p-value for Paired t-test

**: Compared between groups; p-value for Wilcoxon rank sum test

†: Compared between groups; p-value for the generalized linear model adjusted by age, sex, body mass index, smoking, physical activity, alcohol consumption, stress, diet habit, and caffeine intake
